# A comparative study of sampling methods in the detection of esophageal cancer-related microbiota

**DOI:** 10.1128/spectrum.00389-24

**Published:** 2024-07-09

**Authors:** Xia Xue, Siyu Wang, Yi Li, Zhenzhen Liu, Jun Zhang, Ziqing Hu, Chengcheng Fan, Xiaojuan Zhang, Hongle Li, Jun Li

**Affiliations:** 1 Department of Molecular Pathology, Affiliated Cancer Hospital of Zhengzhou University and Henan Cancer Hospital, Zhengzhou, China; 2 Henan Key Laboratory of Helicobacter pylori & Microbiota and Gastrointestinal Cancer, Marshall Medical Research Center, The Fifth Affiliated Hospital of Zhengzhou University, Zhengzhou, China; 3 Department of Endoscopy Center, Affiliated Cancer Hospital of Zhengzhou University and Henan Cancer Hospital, Zhengzhou, China; 4 Department of Applied and Computational Mathematics and Statistics, University of Notre Dame, South Bend, Indiana, USA; 5 Department of Radiation Oncology, Affiliated Cancer Hospital of Zhengzhou University and Henan Cancer Hospital, Zhengzhou, Henan, China; 6 Scientific Research and Discipline Management Office, The Fifth Affiliated Hospital of Zhengzhou University, Zhengzhou, Henan, China­; North Carolina State University, Raleigh, North Carolina, USA

**Keywords:** esophageal microbial structure, sample collection, esophageal environment, microbial diversity, esophageal microbial function

## Abstract

Esophageal cancer (EC) is a multifaceted disease. Our understanding of the involvement of esophageal microbiota in its pathogenesis and progression is limited, which is due to the lack of proper endoscopic sampling methods. Hereby, we conducted a comparative analysis of paired samples obtained through endoscopic brushing and cytosponge, aiming at assessing the feasibility of using cytosponge as a minimally invasive sampling way for studying esophageal microbiota. Our findings suggest that cytosponge sampling yielded significantly superior community richness and diversity compared to endoscopic brushing in both controls (non-cancerous) and EC individuals. The analysis of beta-diversity revealed distinct microbial community pattern in the genus diversity between the two sampling methods, underscoring the importance of selecting appropriate sampling methods to effectively characterize the esophageal microbiota. Specifically, *Lactococcus* and *Serratia* showed higher abundance in the samples collected by endoscopic brushing, while *Alloprevotella* and *Leptotrichia* were more enriched in the samples collected by cytosponge. These differences in dominant microbes were associated with metabolic pathways that particularly were related to host inflammation, such as pyruvate and glucose metabolisms. Notably, the phylogenetic levels of the microbiota indicated varied explanatory power for different detection purposes. This study underscores the substantial impact of sampling method selection on the acquisition of esophageal microbiota associated with the EC development, encompassing considerations of both abundance and diversity. This highlights the significance of selecting an appropriate sampling method for investigating the esophageal microbial status and studying the micro-environment in EC-related individuals.

**IMPORTANCE:**

This study addresses a critical issue in esophageal cancer study by comparing two different sampling methods, endoscopic brushing and cytosponge, for investigating the esophageal microbiota. Our work highlights the suitability of the cytosponge technique as a minimally invasive sampling method for studying the esophageal microbiota and emphasizes the importance of selecting an appropriate sampling method to characterize the microbial community. Our findings have significant implications for advancing the understanding of the role of the esophageal microbiota in cancer development and will inform future research and clinical approaches in this field.

## INTRODUCTION

Esophageal cancer (EC) is an aggressive disease with a poor prognosis due to its insidious onset and asymptomatic development in the early stages (1, 2). The overall 5-year survival rate for EC is around 30% and highly varies among individuals due to diverse clinical symptoms and diagnostic efficacy (3, 4). The development of EC can be driven by multiple factors, that is, genetic background, alcohol and tobacco consumption, human papillomavirus infection, and complex interactions between genetic predispositions and environmental exposures (5, 6). Accumulating evidence suggests that the development of EC is influenced by the composition of the esophageal microbiota (7, 8). However, the intricate relationship between the esophageal microbiota and EC remains incompletely understood due to the multifaceted microenvironmental heterogeneity inherent in individuals with EC. Moreover, it is challenging to discern the dynamics of esophageal microbial structures during EC development, which can be makers for EC diagnosis and treatment. Unraveling the interactions between the esophageal microbiota, the host, and environmental factors is critical for understanding EC development, facilitating preventive measures, and identifying potential therapeutic interventions.

The involvement of esophageal microbiota extends to both the development of and treatment responses of EC patients (9, 10), particularly in radiotherapy, chemotherapy, and targeted therapy (11, 12). However, obtaining samples of esophageal microbiota poses unique challenges due to the delicate nature of the esophageal tissue and the potential for discomfort or injury during procedures (13, 14). Canonical approaches used to collect esophageal microbiota, such as endoscope brushing and interoperative biopsy, are invasive and can only portray the local landscape (15). Intraoperative sampling can offer a less biased depiction of the local microbiota, but it is accompanied with additional issues caused by the anatomical location and surgical context (16). Exploring additional methodologies for the collection of esophageal microbiota is imperative for furthering our understanding of EC concurrence and development.

Cytosponge, also known as EsophaCap, is a medical device primarily used for collecting esophageal exfoliated cells to screen for esophageal diseases, including Barrett’s esophagus (8, 14). Several studies have demonstrated that the effectiveness of cytosponge as a non-invasive method for sampling exfoliated esophageal cells is outstanding compared with other methods (17, 18). In this study, we collected samples from the same individual (non-cancerous or EC patients) by endoscopic brushing (method B) and cytosponge (method C), respectively, followed by 16S rRNA sequencing to compare the esophageal microbial community obtained from these two sampling methods. Our objective is to assess the effectiveness of methods B and C in capturing the diverse microbiota from the same individual, which would provide evidence on the potential utilization of esophageal microbiota for the early diagnosis and precise treatment of EC.

## MATERIALS AND METHODS

### Study participants

A total of 90 patients exhibiting symptoms of esophageal disease, who visited Henan Cancer Hospital (Zhengzhou, China) between November 2021 and April 2022, were included in this study. Patients who had undergone surgical treatment, radiotherapy, or chemotherapy were excluded from this investigation.

### Patients grouping and sample collection

Experimental groups were set using diagnosed EC patients with clinically confirmed esophageal squamous cell carcinoma (ESCC) or esophageal adenocarcinoma (EAC) (*n* = 70), and other esophageal diseases (*n* = 20). Microbiota samples were collected from each patient by method C first, and by method B subsequently. Briefly, the patients were asked to swallow the cytosponge capsule, which dissolved approximately in 5 min to access the lower esophagus and cardia. The capsule, along with its 40 cm long thread, was then gently extracted from the mouth, immersed in the cell preservation solution, and then centrifuged at 4,000 rpm for 15 min. After discarding the sediment, the carefully retained supernatant was thoroughly mixed into a mesh sponge box before storage at −20°C for further analysis. Method B was used to collect esophageal microbiota from the same patient after an interval of over 3 h from Method C. Esophageal mucosal specimens collected by method B were immediately transferred into a sterile preservation solution, stored at −80°C for half an hour, and at −20°C for long-term conservation.

### DNA extractions

Total DNA extraction was performed using the cetyltrimethylammonium bromide (CTAB) method, following the manufacturer’s instructions. The reagent was specifically formulated for efficient retrieval of DNA from minimal sample quantities and has demonstrated efficacy across various bacterial species. Negative control samples were treated with nuclease-free water. Elution buffer was used to extract the total DNA, yielding a final volume of 50 µL, which was then preserved at −80°C until further analysis by PCR amplification test at LC-Bio Technology Co., Ltd in Hangzhou, China.

### PCR amplification and 16S rDNA sequencing

PCR amplification was conducted in a 25 µL reaction mixture composed of 25 ng of template DNA, 12.5 µL of PCR Premix, 2.5 µL of each primer (341F: 5′-CCTACGGGNGGCWGCAG-3′; 805R: 5′-GACTACHVGGGTATCTAATCC-3′). Amplification of prokaryotic 16S rRNA fragments was initiated with an initial denaturation step at 98°C for 30 s, followed by 32 cycles of denaturation at 98°C for 10 s, annealing at 54°C for 30 s, and extension at 72°C for 45 s. A final extension step was carried out at 72°C for 10 min. The size of PCR products was confirmed through electrophoresis using a 2% agarose gel. To ensure the reliability of PCR results, ultrapure water served as a negative control during the DNA extraction. Following PCR, the resulting products underwent purification using AMPure XT beads (Beckman Coulter Genomics, Danvers, MA, USA) and were quantified via Qubit (Invitrogen, Waltham, MA, USA). Amplicon pools then were prepared for sequencing, with the size and quantity of the amplicon library assessed using the Agilent 2100 Bioanalyzer (Agilent, Santa Clara, CA, USA) and the Library Quantification Kit for Illumina (Kapa Biosciences, Woburn, MA, USA), respectively. Sequencing was conducted on the NovaSeq PE250 platform.

### Data analysis

Paired-end reads were initially assigned to their respective samples based on unique barcodes, and then the barcodes and primer sequences were removed through truncation. The pair-end reads were merged into one fastq file using FLASH software. To guarantee high-quality production, clean tags and raw reads were processed to stringent quality filtering using fqtrim (v0.94), following predefined conditions (19). Chimeric sequences filtering was conducted through Vsearch software (v2.3.4) (20). Post-dereplication with DADA2, a feature table and sequence were derived (21). Both alpha diversity and beta diversity were assessed by normalizing sequences to identical counts. For evaluating community diversity within a sample, alpha diversity was measured using five indices: Chao1, Observed species, Goods coverage, Shannon, and Simpson. These calculations were performed using QIIME2 (https://qiime2.org/). Moreover, beta diversity was evaluated to analyze differences between samples (22). Various analytical techniques, including principal component analysis (PCA), principal coordinates analysis (PCoA), non-metric multidimensional scaling (NMDS), and unweighted pair group method with arithmetic mean (UPGMA), were employed to evaluate microbial diversity across distinct environmental communities sampled by different methods from controls and patients. All figures were generated using the R package (v3.5.2). Sequence annotation involved Blast for sequence alignment, and the feature sequences were investigated using the SILVA database.

### Statistical analysis

To compare the observed amplicon sequence variants (ASV) and diversity index between esophageal microbiota sampled from methods C and B, chi-square, Fisher’s exact test, Mann–Whitney *U* test, and Kruskal–Wallis test were conducted. Spearman correlation analysis was performed for the correlation analysis. The statistical analysis and the creation of diagrams were illustrated using R software (v3.6.1, http://www.r-project.org/), and a *P* value of <0.05 was considered statistically significant.

## RESULTS

### Clinical characteristics of the participants

A total of 90 cases, including 71 male patients and 19 female patients, were enrolled in this analysis, among which, 12 cases were finally diagnosed as EAC, 58 were diagnosed as ESCC, and 20 were set to be non-cancerous controls. The average age of the participants was 60.4, 59.8, and 56.9 years for EAC, ESCC, and non-cancerous patients, respectively (Table 1). Based on *t*-test and chi-square statistics, there is no significant difference in the age, smoking, and drinking habits, and body mass index (BMI) comparison between EAC and ESCC groups (*P* > 0.05), while a substantial higher proportion of early-stage disease were observed in ESCC patients than EAC patients (*P* < 0.05, chi-square = 16.2527). To avoid data bias, we conducted random sampling in our pooled sequences, which ensured that our results were representative and reflective of the overall population.

**TABLE 1:** Baseline characteristics of participants

Variables	EAC (*n* = 12)	ESCC (*n* = 58)	Statistical test	Control (*n* = 20)
Average age (year)	60.4	59.8	NS^*a*^	56.9
Male	10	49		12
Female	2	9		8
Current smoking	2	22		5
Current drinking	6	30		6
Average BMI	22	22.9		22.4
Tumor differentiation				
Highly	0	4	Not applicable	/^*b*^
Moderately	2	12		/
Poorly	6	7		/
Moderately-highly	0	2		/
Moderately-poorly	1	5		/
Unknown	2	28		/
Tumor stage			*P* = 0.001	
I	1	22		/
II	2	12		/
III	2	18		/
IV	5	3		/

^*a*^ NS: not significant.

^*b*^ /: not available.

### Amplicon sequencing analysis

We performed V3-V4 16S rRNA sequencing on samples from the same individual (ESCC = 58, EAC = 12, and Control = 20), collected by methods B and C, respectively. The average Q30 score was 89.06 for method B with a guanine (G) and cytosine (C) (GC) content of 53.09%, while for method C, it was 90.19 with a GC content of 51.88%. Both methods yielded valid percentages of reads exceeding 85%, ensuring the reliability of the sequencing data. The number of sequences obtained from each sample was considered sufficient for further analysis. Following amplicon sequencing, we identified a total of 11,771 unique ASVs in method C, compared to 4,849 ASVs in method B. Notably, 4,745 ASVs were found to be shared between methods C and B, indicating commonality in the esophageal microbiota across both sampling methods.

### Alpha diversity across different groups and sampling methods

**FIG 1:**
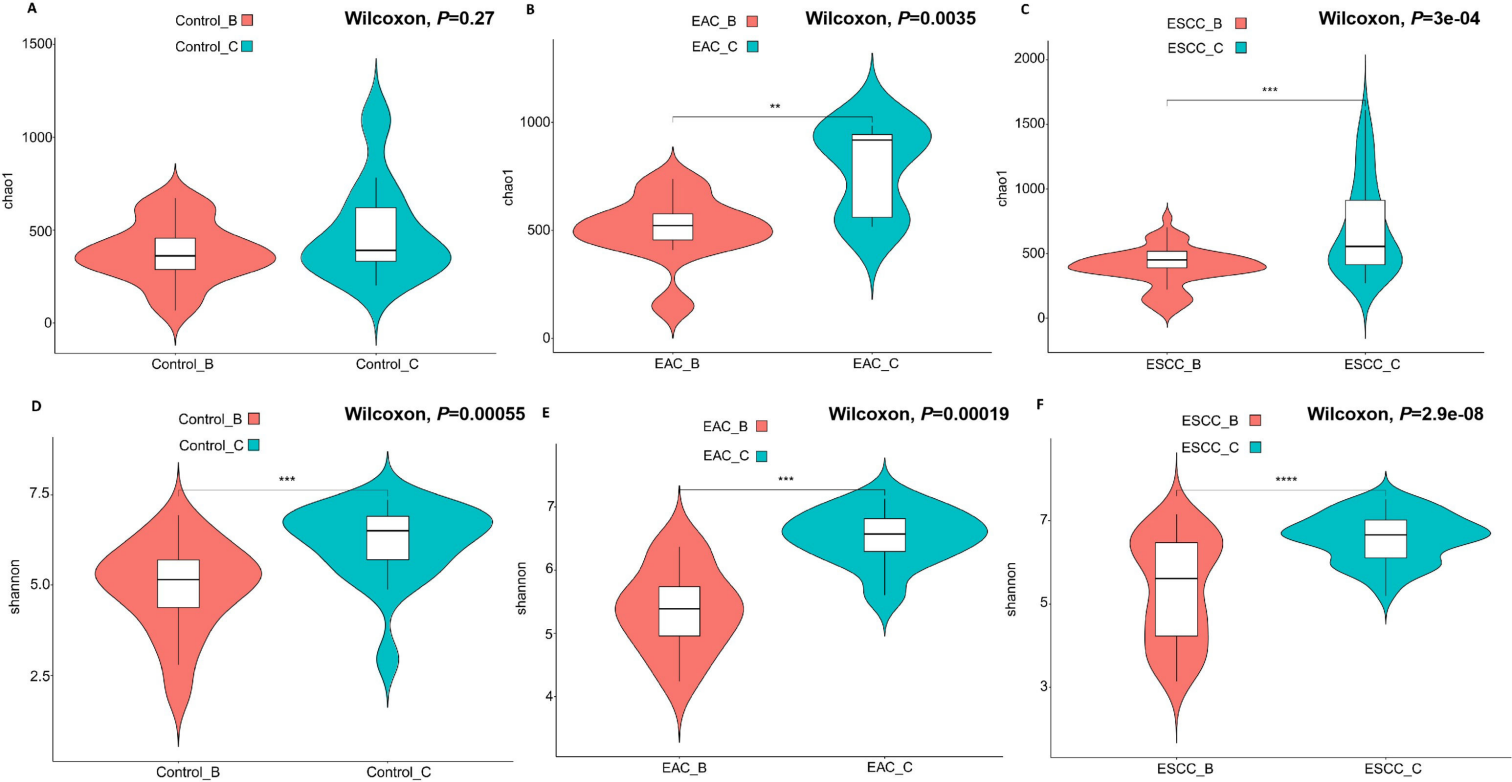
Comparison of alpha diversity, as assessed by ASV, between methods C and B across different cohorts. Community richness analysis based on Chao1 index was performed in the control patients (A), EAC patients (B), and ESCC patients, to make comparisons between method B and method C, respectively. Similar comparisons were presented as Shannon index (D, E, and F). A *P* value of <0.05 was considered statistically significant.

Alpha diversity, reflecting diversity within a specific environment, encompasses species richness, evenness, and sequencing depth. Our analysis showed methods B and C had different performance in community richness and diversity, no matter the samples were collected from EAC/ESCC patients or non-cancerous controls (Fig. 1). In the control group, community richness did not significantly differ from methods B and C (Fig. 1A, *P* = 0.27), while method C exhibited significantly higher community richness of esophageal microbiota (EAC and ESCC) compared to method B (Fig. 1B and C, *P* < 0.05). In parallel, method C demonstrated superior efficacy in esophageal microbiota sampling, evidenced by significantly greater community diversity compared to method B across control (Fig. 1D, *P* < 0.05), EAC (Fig. 1E, *P* < 0.05), and ESCC (Fig. 1F, *P* < 0.05) patients. Diversity analysis suggested that method C outperformed method B in terms of community richness and diversity among the targeted bacteria within the esophageal ecosystem (Table S1).

### Beta diversity across different groups and sampling methods

**Fig 2:**
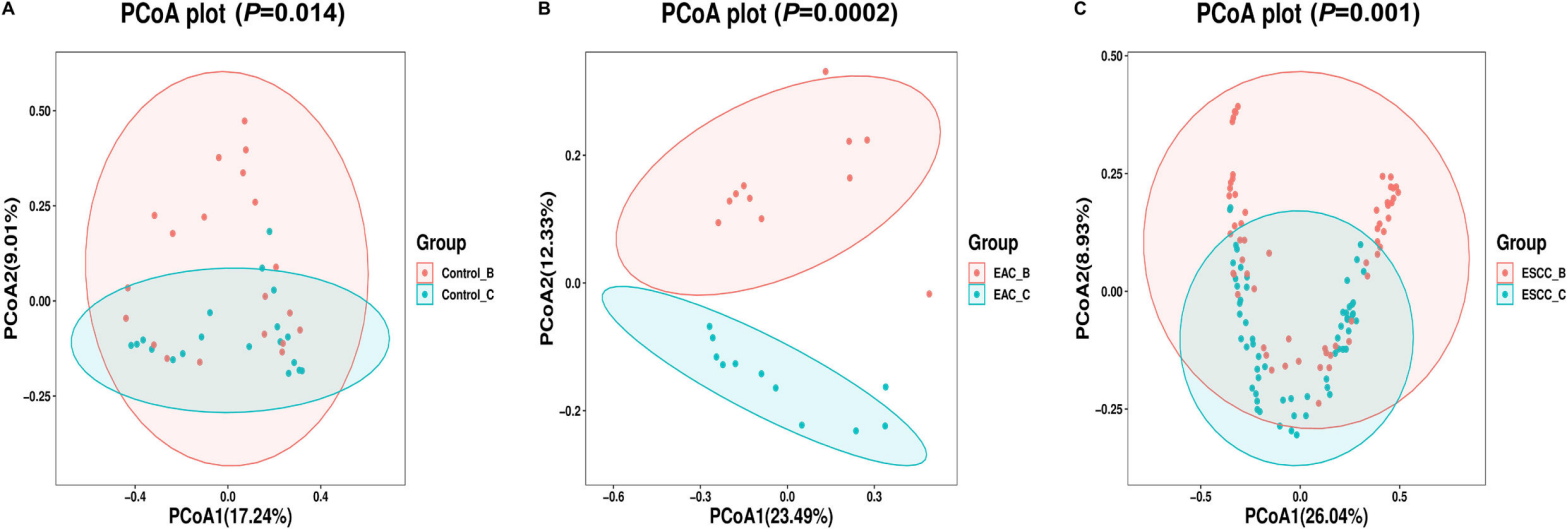
Esophageal bacterial community beta-diversity. PCoA was performed on the esophageal bacterial community at the genus level using sampling methods B and C. (A) Bray–Curtis PCoA comparing ASV from methods C and B in the control patients. (B) Bray–Curtis PCoA comparing ASV from methods C and B in EAC patients. (C) Bray–Curtis PCoA of ASV comparing methods C and B in ESCC patients. Samples obtained by method B were represented in red, while samples obtained by method C are represented in cyan. A *P* value of <0.05 was considered statistically significant. Mann–Whitney *U* test and Wilcoxon single-rank tests were conducted for distribution and variance evaluation. Bray–Curtis dissimilarity was used for calculating distance metrics.

PCoA analysis was performed by comparing the esophageal bacterial communities, sampled by methods B or C, across non-cancerous controls, EAC, and ESCC patients. The clustering pattern of methods B and C in the control group showed minimal variation, suggesting a similar microbial environment among non-EC individuals (Fig. 2A). Notably, in the EAC patients, two distinct clustering patterns were observed, indicating different sampling methods can influence their efficiency for esophageal microbial collection (Fig. 2B). Similarly, for ESCC individuals, the clustering of samples differed significantly between two methods, albeit with some overlapped (Fig. 2C). Additionally, our analysis revealed clear disparities in species diversity between two sampling methods, alongside a notable proportion of shared species, indicating consistent capture of certain microbial species by both methods B and C (Fig. S1). These findings underscore the reliability of our sampling methods in consistently capturing microbial species from EC patients. Our beta-diversity analysis further provides valuable information on the genus-level regarding the differences and similarities between methods C and B, highlighting the importance of selecting appropriate sampling methods for characterizing the esophageal microbiota in diagnosis and treatment settings.

### Community abundance from different sampling methods

**Fig 3:**
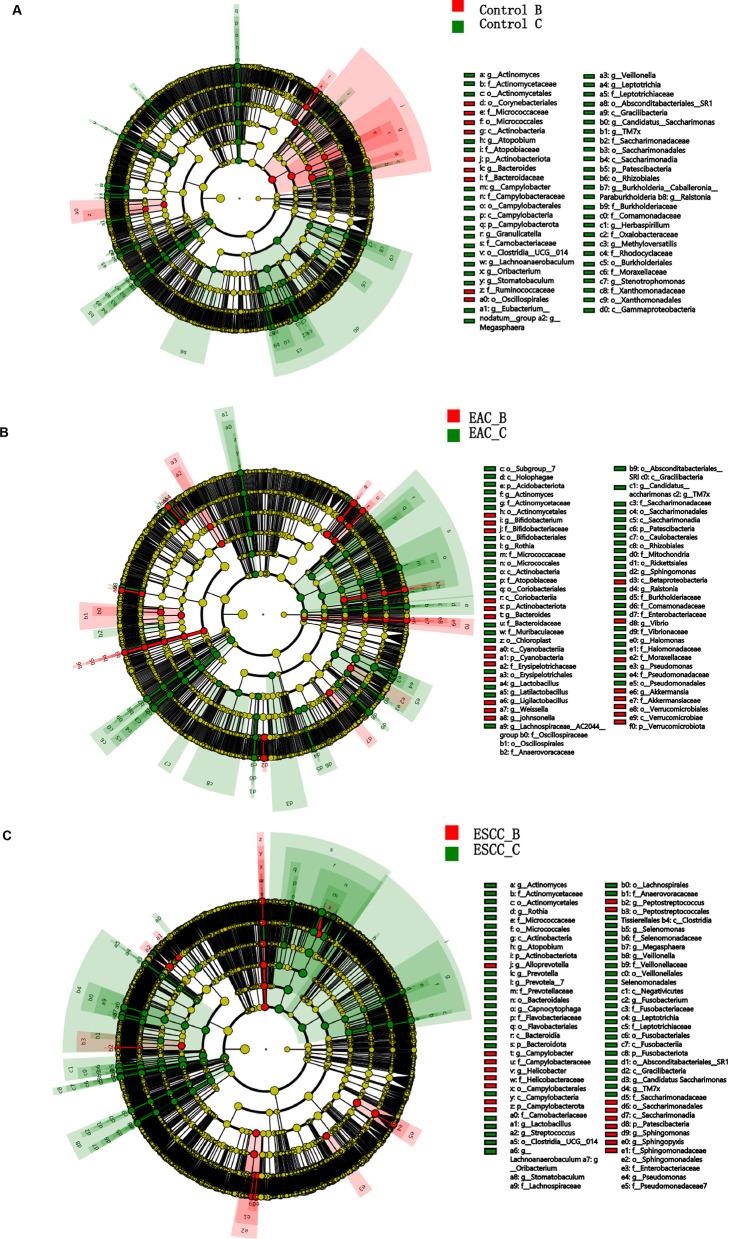
Genus-level taxonomy differences between methods B and C. Variation in relative abundance of microbial communities at the genus level between methods B and C in the non-cancerous control patients (A), EAC patients (B), and ESCC patients (C). Circular layers depict seven classification levels from family to species within phyla. Each node represents a species classification, with node size indicating abundance. Yellow nodes denote species with no significant difference between comparison groups. Nodes in red indicate species with significantly higher abundance in the red group, and the same principle applies to other colors. Gates highlight significant differences, while letters label species with horizontal differences.

Taking into account the ASV counts from methods B and C, we investigated the relative abundance of esophageal microbiota at the genus level using the NT-16S database, with a confidence threshold over 0.7 (Fig. S2). This analysis provided comprehensive community abundance tables at various taxonomic levels, including kingdom, phylum, class, order, family, genus, and species. We herein focused on the genus level to make comparisons between the two methods across three groups (Fig. 3). Our findings indicated minimal taxonomic influence of the methods compared to the EC groups; however, method B demonstrated superior performance for some genera, while method C exhibited suitability for others (Fig. 3A). Notably, EC-associated genus like *Streptococcus*, *Lactococcus*, and *Prevotella* exhibited different abundances between the two sampling methods (Fig. 3B and C). Collectively, method C yielded a higher abundance of esophageal bacteria in EC patients, suggesting its potential suitability for targeting EC-related bacteria, whereas method B might be advantageous in capturing a broader spectrum of microbial diversity.

**Fig 4:**
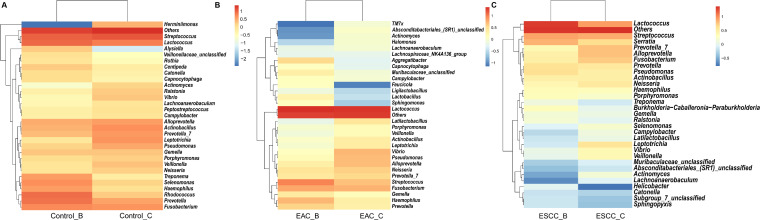
Classification of microbial community based on ASV counts from samples collected by methods B and C. Relative abundance of the top 30 genera sampled by methods C and B in the non-cancerous control patients (A), EAC patients (B), and ESCC patients (C). The genus annotation was indicated next to the heatmaps, and the color gradient from red to blue indicates the relative abundance at the genus level across different groups. The right-side bar in the heatmap denotes the fold change between two different sampling methods.

We also compared the community compositions of the microbiota, collected by methods B and C, respectively, across different groups (Fig. 4). Differential analysis at the genus level between methods B and C identified the top 30 genera with *P* values below 0.05. In the control group, method C exhibited significantly enriched genera compared to method B, with most genera showing weak association with EC (Fig. 4A). In both EAC and ESCC patients, the relative abundance of esophageal bacteria genera sampled by methods C and B differed significantly (Fig. 4B and C). Notably, a similar genus structure was observed in the bacterial communities of EC groups (EAC and ESCC) with both methods, albeit with certain genera showing variation. For example, *Vibrio*, in which *Vibiro cholerae* is notably responsible for diarrheal disease, was more prevalent in samples collected via method C across all groups.

Given the previous evidence supporting the effectiveness of method C in collecting esophageal cells or bacteria at different stages (16, 23), we investigated its utility in ESCC within our study. We designated four stages (T1–T4) in 59 ESCC individuals (T1: 22, T2: 13, T3: 13, T4: 3, missing: 3) and compared the relative abundance of the top 30 genera sampled using method C and B (Fig. S3). *Lactococcus* emerged as the dominant genus sampled by both methods, displaying higher abundance at T4 and lower at T2. Additionally, TM7x and *Absconditabacteriales* were exclusively collected as top dominant genera via method C, suggesting a potential association within the esophagus or gastrointestinal tract.

**Fig 5:**
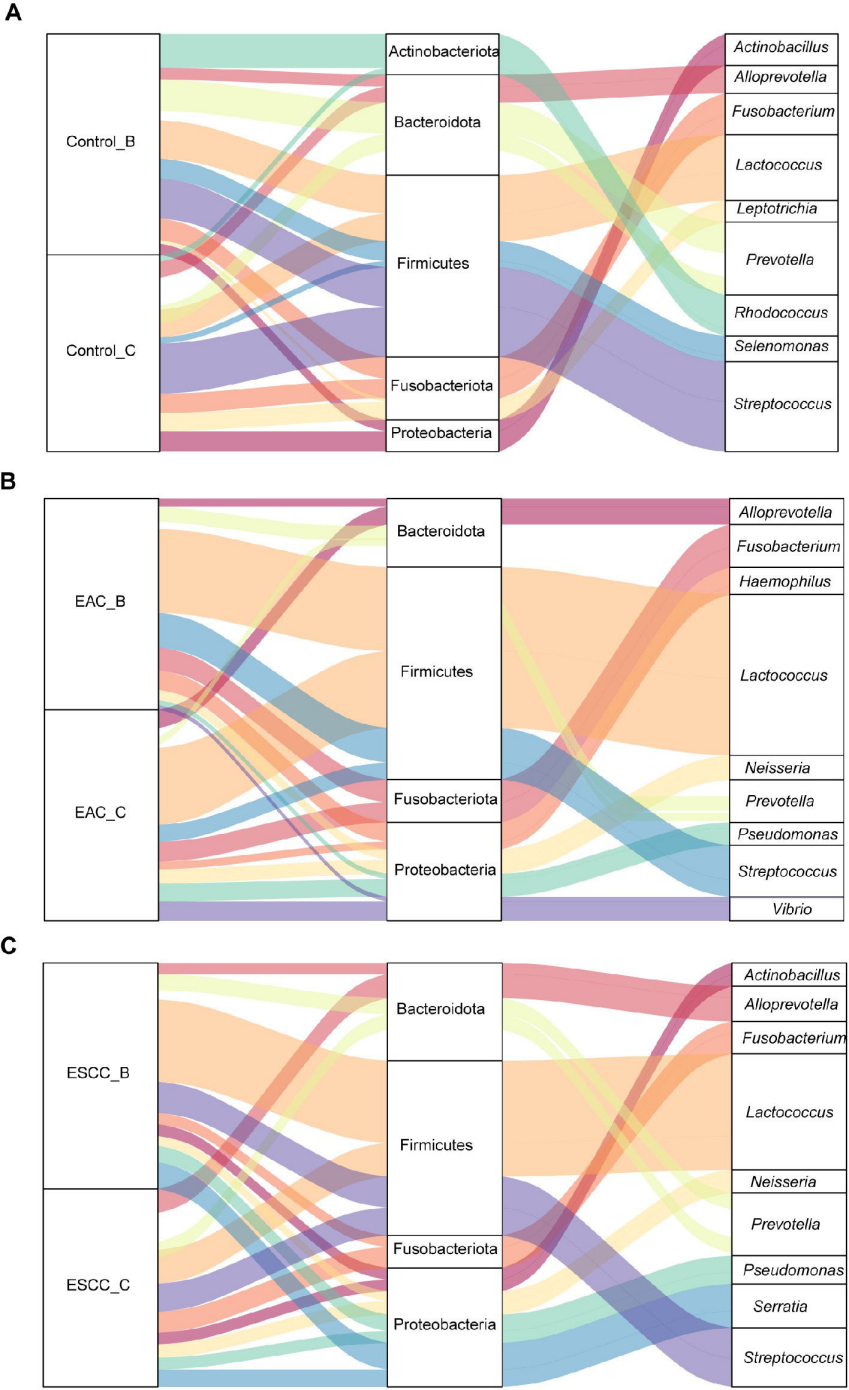
The taxonomy abundance of microbial communities at both the phylum (middle) and genus levels (right), categorized by different sample types (left). It presents species annotation information, correspondence, and proportion, offering insights into the two key levels in microbial diversity research. The labels A, B, and C represent the Control, EAC, and ESCC samples, respectively. A broader segment signifies higher abundance, while a narrower segment denotes lower abundance.

The Sankey plot analysis was employed to visualize the relative abundance of microbial communities at both the phylum and genus levels, corresponding to methods B and C (Fig. 5). Phylum Actinobacteriota was only identified in the control group and was more prominently collected by method B (Fig. 5A). Our findings unveiled the presence of four primary phyla in EC groups (Fig. 5B and C), namely *Bacteroidota*, *Firmicutes*, *Fusobacteriota*, and *Proteobacteria*, observed in both methods B and C. However, there were notable variations in the proportions of each phylum and genus between the two sampling methods. Method B showed a higher efficiency in collecting *Firmicutes* compared to method C, while method C exhibited superior efficiency in capturing *Bacteroidota*. These discrepancies in phylum proportions highlight the importance of selecting an appropriate sampling method based on the target phyla of interest. Remarkably, at the genus level, the difference in collection efficiency between the two methods may not be as pronounced as at the phylum level. The analysis at the genus level suggests that both sampling methods are relatively effective in capturing microbial diversity within this taxonomic stratum. To comprehensively assess the different genera collected by each method, we compared the ASV signals between methods B and C. Our findings indicate that *Lactococcus* was more prominently extracted by method B, being the dominant genus in esophageal microbiota, while method C showed better sensitivity to other genera with relatively lower abundance.

### Function annotation

**Fig 6:**
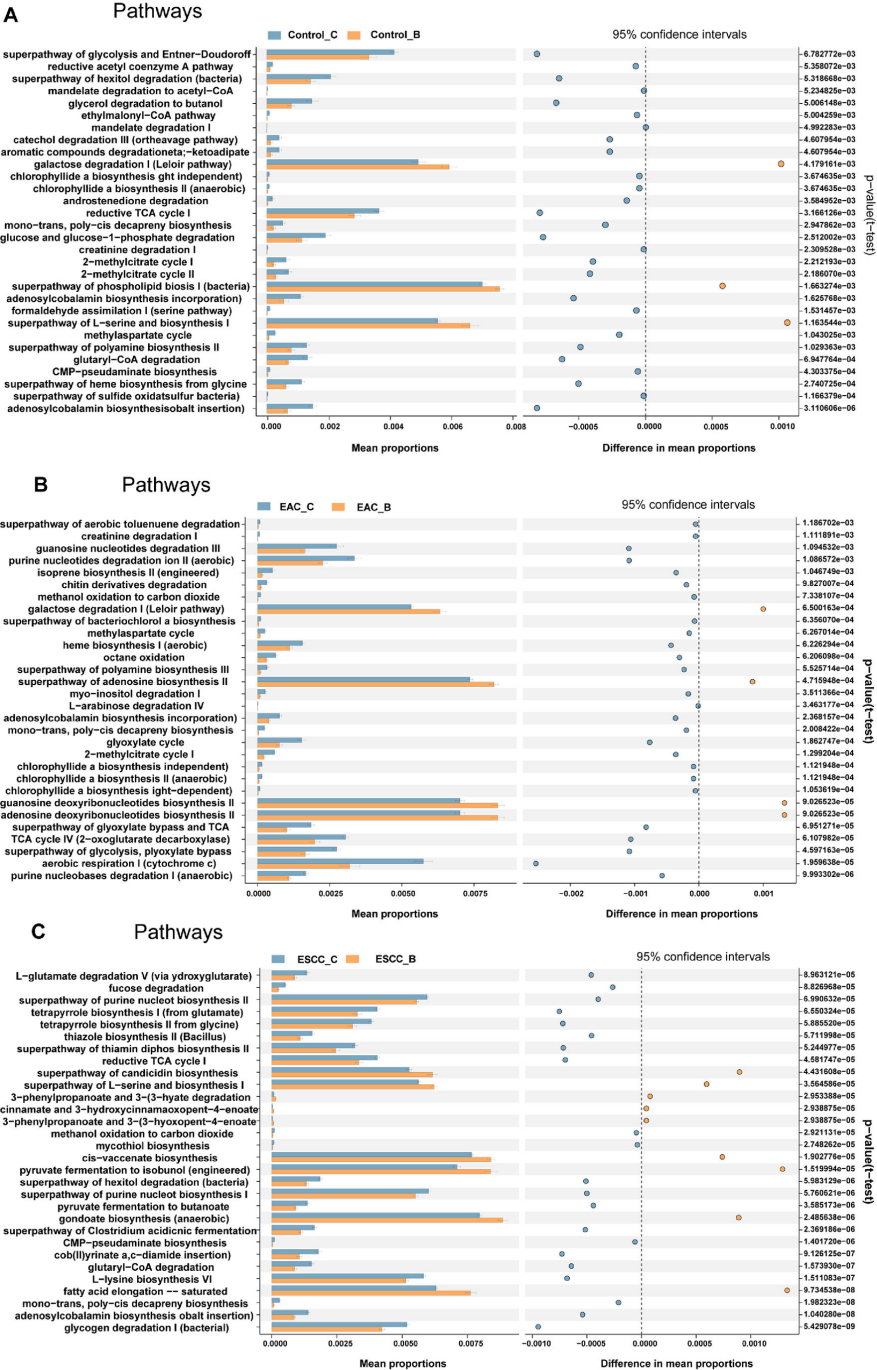
Function annotation of esophageal microbiota sampled by methods B and C. Panels A, B, and C illustrate KEGG annotation based on PICRUSt2 from ASV obtained via methods B and C in the control patients, EAC patients, and ESCC patients, respectively. Top 30 function annotations with a significance level of *P* < 0.05 (*t*-test) in pairwise comparisons, were present, and the confidence interval is set at 95%.

The STAMP analysis yielded significant results, identifying the top 30 functions with significant differences between methods B and C across three groups (*P* < 0.05, Fig. 6). These observed disparities in functions, supported by a 95% confidence interval, provided initial evidence about the association of bacterial populations sampled via methods B and C with relevant functional pathways. Notably, most pathways linked to host inflammation showed significantly differential expression between methods B and C (Fig. 6B and C, *P* < 0.05), including pathways related to pyruvate metabolism and fatty acid elongation. The distinct functional differences within these pathways suggest potential variations in host-microbiota interactions between the two sampling methods or within different micro-environments. Furthermore, certain pathways, such as glycogen degradation, were found to be associated with the relative abundance of specific bacterial species, suggesting the composition of the esophageal microbiota, as captured by different sampling methods, may influence microbiota functionality within the host. The correlation between bacteria and their associated pathways within the host could be influenced by factors such as diet, genetic background, and the different stages of EC in patients. These findings underscore the complexity and multifaceted nature of the interactions between esophageal microbiota and the host, potentially impacting disease development and progression.

## DISCUSSION

**FIG 7:**
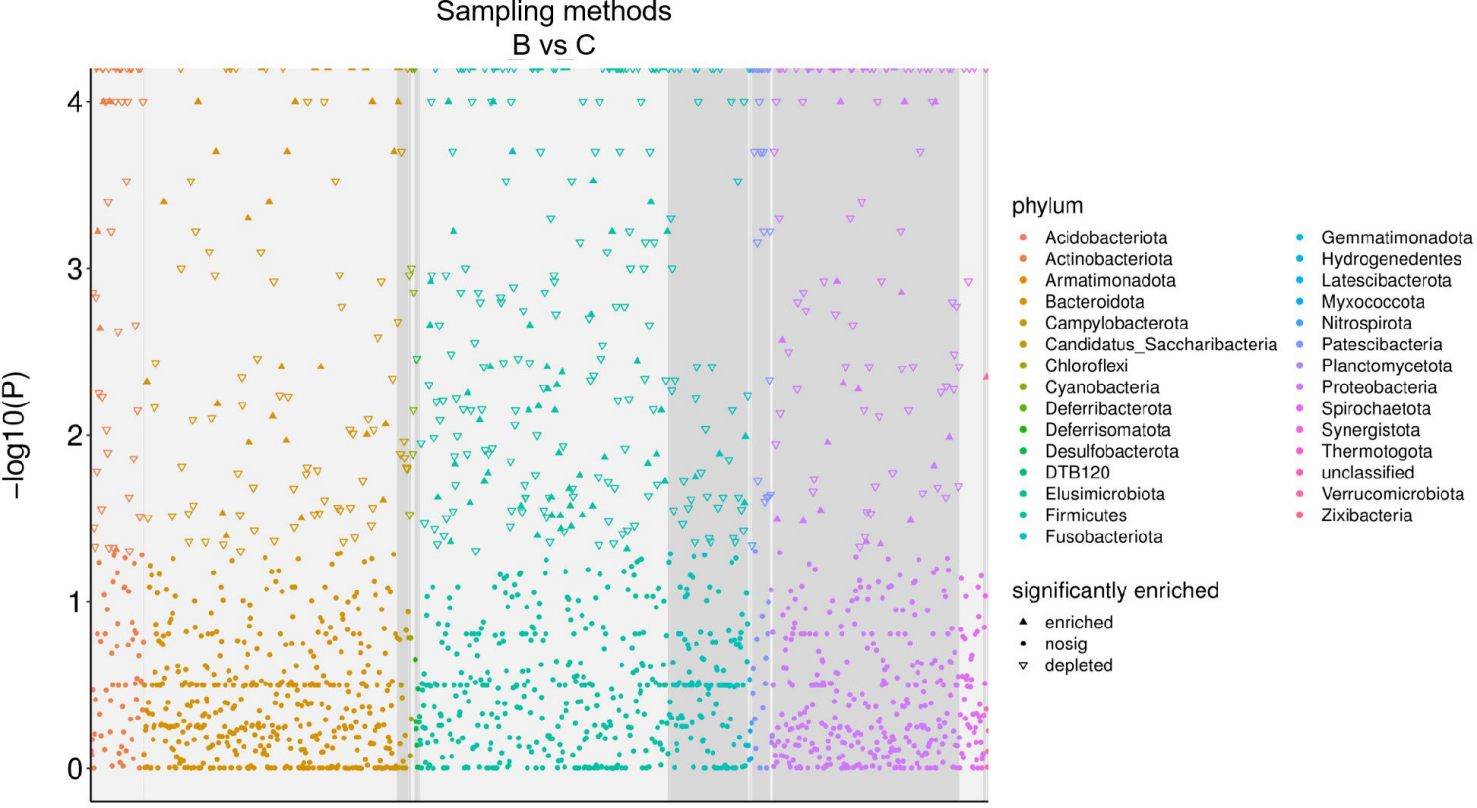
Differences in ASV abundance analyzed by using pairwise comparison methods. Enriched (upper triangle) denotes significant upregulation, depleted (lower triangle) denotes significant downregulation, and nosing (dot) indicates no significant difference. Different colors were used to differentiate the phylum-level species corresponding to each ASV.

The esophagus is a relatively sterile environment and has been found to harbor a diverse microbial community (24, 25). Although the esophageal microbiota is less well-studied compared to the gut microbiota, understanding the bacteria that inhabit the esophagus would be beneficial for early diagnosis and precise personal treatment of EC. The present study employed invasive (method B) and minimal-invasive sampling methods (method C) on 70 EC patients to evaluate their collecting coverage and efficiency in collecting esophageal microbiota (Fig. 7). Different from previous comparisons between esophagus biopsy and swab sampling, which showed similar microbial characteristics (26), we found that method C collected more ASVs than method B. This indicates that the diversity and richness of the esophageal microbiota sampled by method C were higher than those by method B, regardless of the group. Notably, for certain species of the esophageal microbiota, method B showed broader coverage. The top five genera in terms of relative abundance from both methods include *Lactococcus*, *Streptococcus*, *Fusobacterium*, *Alloprevotella*, and *Prevotella*, which differed from previous observations (26). This disparity might be attributed to the individual heterogeneity of EC patients across different cohorts. At the phylum level, the top dominant phyla included *Bacteroidota*, *Firmicutes*, *Fusobacteriota*, and *Proteobacteria*, consistent with findings from other investigations (16). Therefore, comparing the esophagus microbial structure at a higher hierarchical level may be more feasible for signature determination. We annotated the microbiota to the genus level to observe individual microbial diversity among various EC patients under different conditions, providing a clue that method C could be a promising strategy for particular stages of EC patients or specific symptoms/positions, compared to method B.

Previous studies suggest an association between certain bacteria and EC, such as *Helicobacter pylori* and *Streptococcus anginosus* (27, 28). *H. pylori*, a pathogenic bacterium that primarily colonizes the stomach, has been reported to be potentially linked to EAC (27). Meanwhile, *S. anginosus* is implicated in the initiation and progression of ESCC through its ability to produce carcinogenic metabolites and induce chronic inflammation in the esophageal mucosa; however, disparate results have been observed in different EC cohorts (28, 29). As the mechanisms and causal relationship behind this association remain incompletely explained, it is important to investigate the esophageal microbiota under complex conditions, along with studying their metabolic functions involved in esophageal carcinogenesis. Our investigation revealed that the genus *Streptococcus* showed relatively high abundance in samples collected by method B, particularly in ESCC group. *Streptococcus* is one of the dominant bacteria in the esophagus and has been reported to be related to EC occurrence. *Prevotella* and *Veillonella* are genera commonly found in both the oral cavity and esophageal microbiota, which can be more effectively collected better by method C in both EAC and ESCC groups. These bacteria are involved in carbohydrate metabolism and the breakdown of complex polysaccharides, alternation of which might contribute to EC development (7, 30). Additionally, *Streptococcus* and *Veillonella* can tolerate acidic environments during episodes of gastroesophageal reflux, making them dominant in the esophagus (27, 29, 30). Changes in this preference could serve as potential microbial markers for condition changes during EC development of different types of EC. It is noteworthy that the esophageal microbiota can vary significantly among different EC patients, evidenced by comparisons among the control, EAC, and ESCC groups. Our investigation into two sampling methods lays the groundwork for elucidating the pattern of the esophageal microbiota under different conditions for EC patients, as well as in different tumor stages.

The study of the esophageal microbiota has emerged as an essential research field for precision medicine in EC. Various sampling methods have been employed to investigate the esophageal microbiota’s relation to EC. Factors affecting the sampling methods, including invasiveness, potential contamination, and the depth of microbial sampling, bring diffrerent pros and cons for each sampling strategy. The cytosponge (method C) is designed to collect cells and associated microbiota from the entire length of the esophagus. In contrast, the endoscopic brushing (method B) technique primarily targets the mucosal surface at the site of endoscopic examination, which might miss or underrepresent microbial communities present in other esophageal regions. This differential spatial sampling could lead to the enrichment of specific taxa, such as the higher abundance of *Lactococcus* and *Serratia* observed from the method B, and the increased prevalence of *Alloprevotella* and *Leptotrichia* in the samples from the method C. Furthermore, the mechanical forces involved in each sampling method may also contribute to the observed microbial community differences. The gentle sponge-like action of the method C can potentially capture a more comprehensive representation of the esophageal microbiota, including both surface-adherent and loosely associated microbes. The method B, on the other hand, can selectively dislodge and collect microbes firmly attached to the mucosal surface, potentially missing the more loosely associated community members. Consistently, we observed that method C provides a more comprehensive description of esophageal microbiota diversity, while method B tends to collect certain bacteria families, such as *Lactococcus*, more heavily in both types of EC. Furthermore, the metabolic pathways of the esophageal microbiota are better annotated in samples collected by method C, offering greater explanatory power regarding the interaction between esophageal microbiota and EC development. The choice of sampling method thus depends on specific research objectives, study design, and available resources. Method C, being a non-invasive sampling approach covering both the esophagus and part of the gastric region, raises concerns about potential impact on the results of the non-EC individuals. These concerns can be addressed through study design and further analysis, such as filtering out gastric-specific microbes based on the previous work, or by sequencing gastric microbiota from the same individuals to screen out oral ones. Given the close anatomical proximity between the oral cavity and the esophagus, it is reasonable to assume that the oral microbiota may have an impact on the composition and function of the esophageal microbiota. However, it is important to note that while the oral microbiota may contribute to shaping the esophageal microbiota, the esophagus also possesses its unique microbial signatures. Therefore, it is important to note that the field of esophageal microbiota research is still evolving, and further studies are needed to better understand the role of the esophageal microbiome in health and disease, for instance, longitudinal studies tracking microbial dynamics and functional interactions in digestive tract are needed.

### Conclusion

This study highlights significant differences in the esophageal microbiota related to EC and other gastrointestinal diseases when comparing two sampling methods. Cytosponge demonstrated superior performance in detecting a wide range of esophageal microbiota compared to endoscopic brushing in both ESCC and EAC. As a more efficient and appropriate sampling strategy, cytosponge provides valuable insights into the microbial community of EC patients. Our findings contribute to a better understanding of the esophageal microbiota and its potential role in EC development and progression of EC, as well as related conditions. By identifying differences in diversity and abundance of the esophageal microbiota between the two methods, this study offers crucial information for future investigations in this field. Furthermore, the study emphasizes the importance of carefully selecting the appropriate sampling method to accurately characterize the esophageal microbiota in specific research and clinical settings.

## Data Availability

The data are available from the corresponding author on reasonable request. The raw reads in this work were deposited on NCBI (https://submit.ncbi.nlm.nih.gov) with the Project ID PRJNA1071795.

## SUPPLEMENTAL MATERIAL

Fig. S1 spectrum.00389-24-s0001.tif The Nonmetric Multidimensional Scaling from method B and C.

Fig. S2 spectrum.00389-24-s0002.tif The relative abundance of esophageal microbiota at the genus level using the NT-16S database with a confidence level greater than 0.7.

Fig. S3 spectrum.00389-24-s0003.tif The microbial community from methods B and C based on ASV counts at different ESCC stages (T1-T4).

Supplemental material spectrum.00389-24-s0004.docx Figure legends.

Table S1 spectrum.00389-24-s0005.xlsx Data for alpha-diversity.

ASM does not own the copyrights to Supplemental Material that may be linked to, or accessed through, an article. The authors have granted ASM a non-exclusive, world-wide license to publish the Supplemental Material files. Please contact the corresponding author directly for reuse.
