# Supplementary figures and images for: A comparative study of sampling methods in the detection of esophageal cancer-related microbiota
Source: Microbiol Spectr. 2024 Jul 9;12(8):e00389-24. doi: 10.1128/spectrum.00389-24 (PMC11302015; doi:10.1128/spectrum.00389-24)

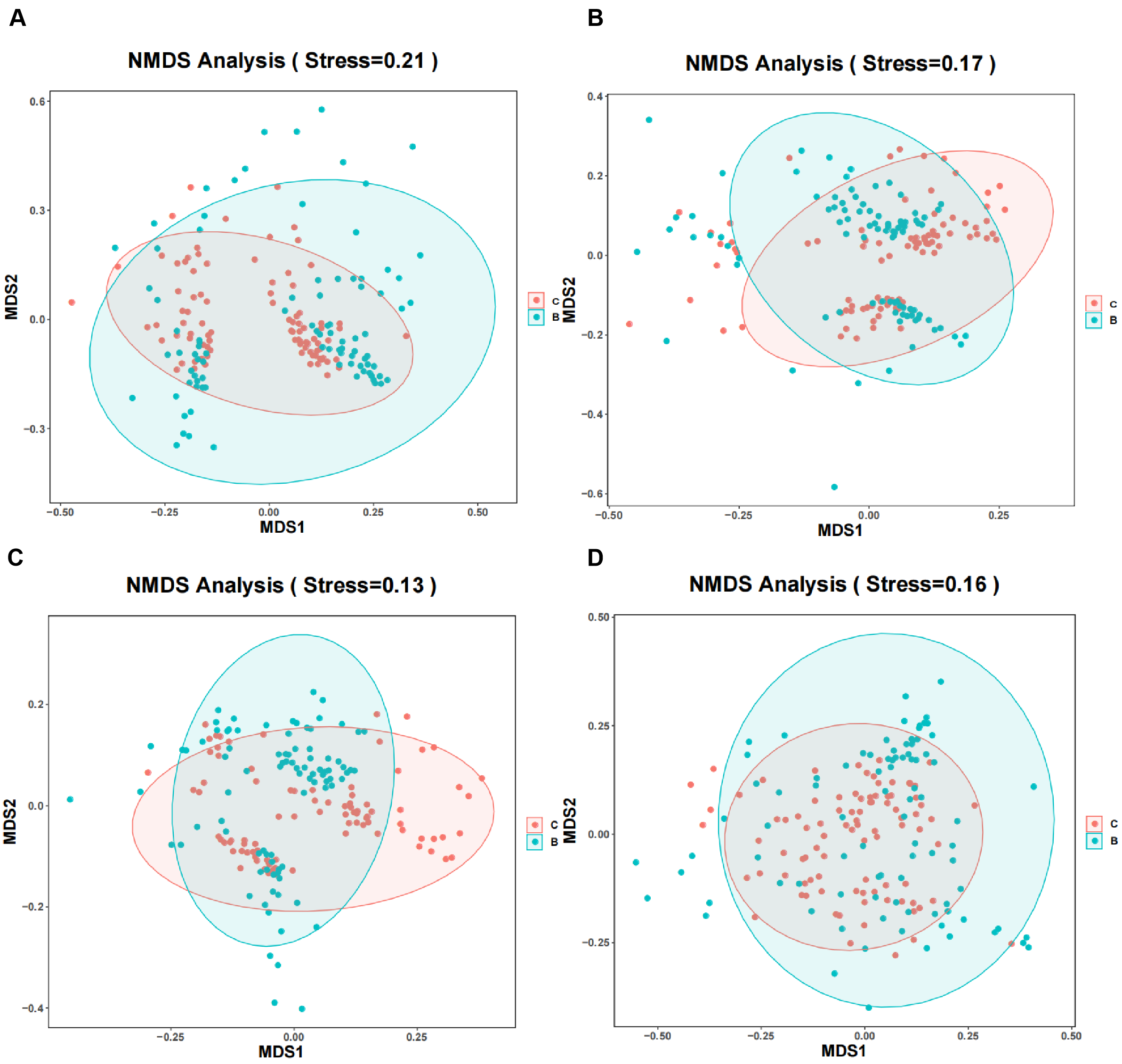

Supplement: Fig. S1 — The Nonmetric Multidimensional Scaling from method B and C. [file spectrum.00389-24-s0001.tif]

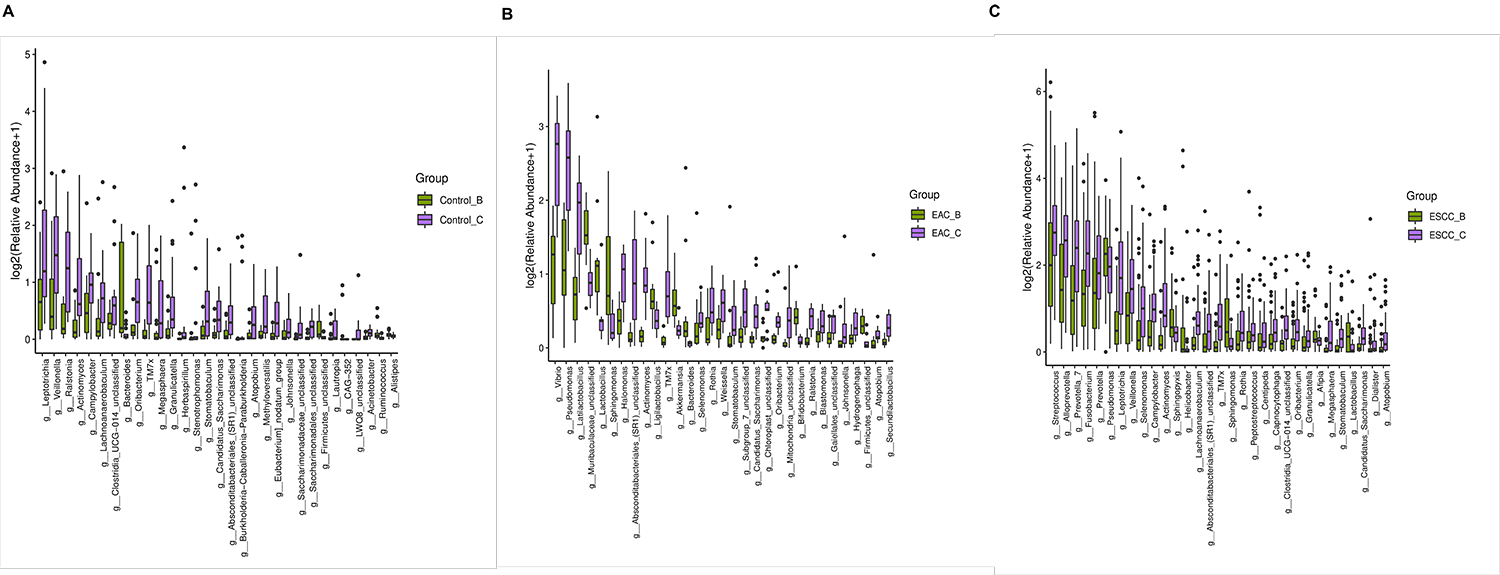

Supplement: Fig. S2 — The relative abundance of esophageal microbiota at the genus level using the NT-16S database with a confidence level greater than 0.7. [file spectrum.00389-24-s0002.tif]

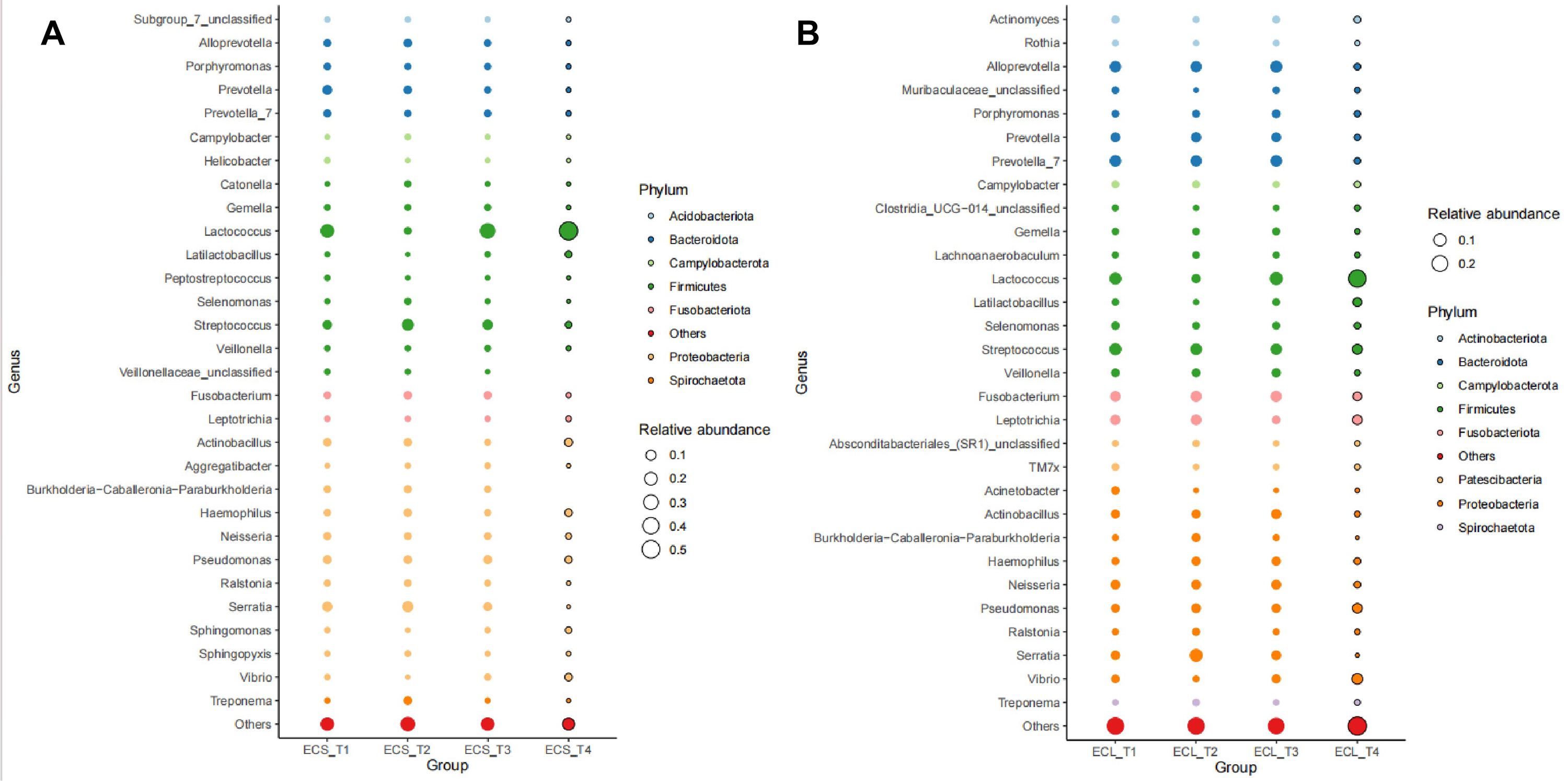

Supplement: Fig. S3 — The microbial community from methods B and C based on ASV counts at different ESCC stages (T1-T4). [file spectrum.00389-24-s0003.tif]
