# Supplementary material for: A comparative study of sampling methods in the detection of esophageal cancer-related microbiota
Source: Microbiol Spectr. 2024 Jul 9;12(8):e00389-24. doi: 10.1128/spectrum.00389-24 (PMC11302015; doi:10.1128/spectrum.00389-24)
Supplement: Supplemental material — Figure legends. [file spectrum.00389-24-s0004.docx]

**Supplementary figure 1:** The Nonmetric Multidimensional Scaling from method B and C. A: The distance calculated by Bray-Curtis and stress = 0.21; B: The distance calculated by Jaccard and stress = 0.17; C: The distance calculated by unweighted_unifrac and stress = 0.13; D: The distance calculated by weighted_unifrac and stress = 0.16. The stress value in NMDS analysis represents the normalized sum of squared differences between the original dissimilarities and the distances in the NMDS plot (0-1). A lower stress value indicates a better fit or representation of the original dissimilarities in the NMDS plot, suggesting a more accurate visualization of the underlying structure of the data.

**Supplementary figure 2:** The relative abundance of esophageal microbiota at the genus level using the NT-16S database with a confidence level greater than 0.7. A: In the control group, the relative abundance of top 30 genus sampled by the method B and C; B: In esophageal adenocarcinoma (EAC) group, the relative abundance of top 30 genus sampled by the method B and C; C: In esophageal squamous cell carcinoma (ESCC) group, the relative abundance of top 30 genus sampled by the method B and C.

**Supplementary figure 3:** The microbial community from methods B and C based on ASV counts at different ESCC stages (T1-T4). A: Based on method C, the relative abundance of top 30 genus sampled at T1, T2, T3, and T4 stages (ECL_T1-T4). B: Based on method B, the relative abundance of top 30 genus sampled at T1, T2, T3, and T4 stages (ECS_T1-T4). The figure shows the genus annotation information, relative abundance (circle size) at the genus level in different sample groups, and the annotation information (circle color) for the corresponding phyla of the genus.
